# Endometrial Repair and Regenerative Medicine: From Broad-Spectrum Regeneration to Precision Intervention

**DOI:** 10.1007/s43032-026-02150-3

**Published:** 2026-07-27

**Authors:** Mengqi Xie, Yungui Cao, Jiangping Tang, Fang Li, Juan Deng

**Affiliations:** Department of Gynecology, Shanghai Jiading District Maternal and Child Health Care Hospital, Shanghai, 201800 China

**Keywords:** Endometrial injury, Mesenchymal stem cells, Platelet-rich plasma, Extracellular vesicles, Regenerative medicine

## Abstract

Endometrial injury-related disorders, including intrauterine adhesions, thin endometrium, and chronic endometritis, are a major cause of female infertility. Conventional therapeutic approaches, primarily hormone therapy and surgical interventions, show limited effectiveness in patients with moderate to severe endometrial damage. In this context, regenerative medicine has emerged as a promising direction to overcome current therapeutic limitations and promote functional reconstruction of the endometrium. A comprehensive literature search was conducted across PubMed, Web of Science, Embase, and Scopus to identify relevant studies on endometrial repair and regenerative medicine. The search covered publications from January 2000 to August 2025. The following keyword combinations and MeSH terms were used: (“endometrial repair” OR “endometrial regeneration” OR “intrauterine adhesion” OR “Asherman syndrome” OR “thin endometrium” OR “chronic endometritis”) AND (“mesenchymal stem cells” OR “platelet-rich plasma” OR “exosomes” OR “extracellular vesicles” OR “biomaterials” OR “hydrogel” OR “scaffold” OR “regenerative medicine”). Both basic science investigations and clinical studies were included to provide a comprehensive overview of current developments in the field. To minimize the risk of omission, the reference lists of included articles and relevant reviews were manually screened, and potentially pertinent studies were further evaluated. Inclusion criteria: (1) Original research articles or meta-analyses; (2) Focus on therapeutic interventions for endometrial regeneration; (3) Human clinical studies or mammalian animal models. Exclusion criteria: (1) Non-English articles, letters, conference proceedings, etc.; (2) Purely descriptive studies of endometrial physiology without therapeutic intervention, articles lacking quantitative data or sufficient methodological details. The screening process followed the PRISMA 2020 guidelines (Fig. 1). After removing duplicates, 1936 records were screened by title and abstract, and 605 full-text articles were assessed for eligibility. Finally, 102 studies were included in this qualitative synthesis. This review provides a systematic synthesis of recent advances in cell-based therapies, cell-free approaches, and bioengineering strategies for endometrial repair. Evidence derived from different sources of mesenchymal stem cells, including bone marrow, umbilical cord, and endometrium, is comparatively evaluated alongside platelet-rich plasma and extracellular vesicles, with an emphasis on hierarchical assessment of evidence levels. Several critical issues are further examined. Current stem cell-based interventions are largely characterized by a broad reparative profile, yet precise targeting of key molecular mechanisms remains insufficient. To date, no studies have demonstrated the capacity to directly reverse suppression of signaling pathways such as the GZMA-PARD3 axis or to restore depleted regenerative stromal subpopulations, including IGFBP3⁺ cells. Clinical investigations of platelet-rich plasma exhibit substantial heterogeneity, which appears to stem mainly from the absence of standardized preparation protocols and the frequent reliance on surrogate endpoints, particularly endometrial thickness. However, the predictive value of endometrial thickness for live birth is limited, and its use as a primary endpoint may overestimate clinical benefit. Therefore, emphasis should shift toward patient-centered outcomes such as live birth rate. Concerns also arise regarding the degradation kinetics of biomaterials, as mismatches between material resorption and the cyclical regenerative dynamics of the endometrium may increase the risk of secondary adhesions. In addition, the actual delivery efficiency of microneedle-based systems within the enclosed and humid uterine environment has not yet been fully clarified. Based on this critical appraisal, we propose a translational framework that links mechanistic discovery with precision intervention. The importance of long-term follow-up is emphasized, with live birth rate regarded as a more clinically meaningful primary endpoint for evaluating therapeutic efficacy in endometrial regenerative medicine. Endometrial regenerative medicine is transitioning from empirical repair toward mechanism-informed reconstruction. Current evidence is dominated by short-term (< 12 months), small-sample (*n* < 50) exploratory studies with high interventional heterogeneity. Clinical translation will require unified preparation standards, individualized treatment strategies, and well-designed multicenter randomized controlled trials with live birth rate as the primary endpoint and minimum 2-year follow-up.

**Supplementary Information:**

The online version contains supplementary material available at https://doi.org/10.1007/s43032-026-02150-3.

## Introduction

 The structural integrity and functional competence of the endometrium are fundamental prerequisites for successful embryo implantation and the maintenance of pregnancy. Pathological conditions such as intrauterine adhesion (IUA), thin endometrium (TE), and chronic endometritis (CE) can compromise the basal layer of the endometrium and are frequently accompanied by fibrotic remodeling, reduced glandular density, insufficient vascular perfusion, and dysregulated expression of receptivity-related molecules. These alterations are closely associated with adverse reproductive outcomes, including infertility, recurrent pregnancy loss, and abnormal placentation [1].

Impaired endometrial repair following injury is regarded as a central pathological feature of intrauterine adhesion (IUA), and the condition often arises secondary to intrauterine surgery, infection, radiotherapy, or tuberculosis. Although the underlying mechanisms have not yet been fully elucidated, accumulating evidence indicates that multiple processes jointly contribute to fibrotic progression, including persistent inflammatory responses, abnormal extracellular matrix (ECM) deposition, epithelial-mesenchymal transition, as well as dysregulation of autophagy and ferroptosis [2]. At the molecular level, fibrogenesis is governed by a network of interacting signaling pathways, among which the TGF-β/Smad axis is considered a pivotal regulator [3]. TGF-β1 has been shown to drive the differentiation of endometrial stromal cells (ESCs) toward a fibroblast-like phenotype, thereby promoting excessive accumulation of ECM components such as collagen and fibronectin. Other pathways, including Wnt/β-catenin [4], Hippo/YAP, JAK-STAT, PI3K/Akt, NF-κB, and cGAS-STING [5], are also implicated in this regulatory framework and collectively shape the profibrotic signaling network in IUA. In parallel, the endometrial microenvironment in patients with IUA is characterized by elevated levels of pro-inflammatory cytokines, including IL-1β, TNF-α, IFN-γ, IL-6, and IL-17 A, which further sustain inflammatory activation and tissue remodeling [6, 7].

Thin endometrium (TE) is generally defined as a condition in which the endometrial thickness fails to reach the physiological threshold despite adequate estrogen stimulation, commonly measured as < 7 mm during the peri-ovulatory or secretory phase, and < 5 mm in severe cases. Such insufficient endometrial development is associated with a markedly reduced probability of embryo implantation [8]. Recent single-cell transcriptomic analyses suggest that the underlying basis of thin endometrium lies in a disrupted balance between stromal regeneration and immune microenvironmental repair, with molecular and cellular dysregulation serving as the primary mechanism driving this pathophysiological process [9].

A reduction in regenerative IGFBP3⁺ Stromal-1 cells has been observed, whereas profibrotic Stromal-2 populations appear to expand, jointly promoting extracellular matrix remodeling dominated by collagen deposition. Concurrent alterations in immune cell composition have also been reported. Natural killer (NK) cells tend to decrease in abundance and exhibit a pro-inflammatory phenotype. T cells show a functional shift from immune regulation toward participation in ECM remodeling. Macrophages are more frequently polarized toward a profibrotic phenotype and are often accompanied by disturbances in lipid metabolism. Cell Chat-based analyses have further indicated that suppression of the GZMA-PARD3 and APOE-TREM2 signaling axes may serve as key drivers of the dysregulated repair process described above. In parallel, excessive activation of adhesion-related and collagen-associated pathways appears to underpin the fibrotic-inflammatory cascade that characterizes the pathological microenvironment [10]. These recently identified molecular targets provide a theoretical foundation for the development of more specific therapeutic strategies aimed at endometrial repair. However, studies designed to achieve precise intervention at the level of defined cellular subpopulations or specific signaling axes remain scarce, and targeted therapeutic approaches in this context are still largely underexplored. The gap between mechanistic discovery and clinical application has become a major obstacle for the field to move beyond descriptive observation toward precision intervention.

Chronic endometritis (CE), characterized as a state of persistent inflammation, disrupts the immune microenvironment required for maternal-fetal tolerance by altering both the composition and functional properties of local endometrial immune cells, including uterine natural killer (uNK) cells and macrophages. This immunological imbalance has been implicated as an important contributing factor to recurrent implantation failure and pregnancy loss, with reported recurrence rates ranging from 9.3% to 67.6% [11]. Owing to its often subtle or nonspecific clinical manifestations, CE remains underdiagnosed in routine practice. Investigations into CE-associated fibrotic mechanisms have revealed that, compared with healthy controls, the endometrium of reproductive-aged patients with histologically confirmed CE exhibits increased mast cell (MC) activation and enhanced tryptase secretion. These activated MCs may influence the polarization of monocytes toward M1 or M2 macrophage phenotypes through paracrine and juxtacrine signaling, thereby reshaping the local immune cell network and facilitating region-specific pro-inflammatory or profibrotic responses. Interactions between MCs and CD8⁺ T cells have also been implicated in augmenting cytotoxic activity, which may further contribute to the progression of fibrotic remodeling [12]. Therapeutically, modulating MC activity or their secretory profiles may represent a strategy for preventing and treating CE-related fibrosis. Emerging evidence indicates that attenuation of inflammatory responses and restoration of immune microenvironmental homeostasis promote functional endometrial repair [13].

In clinical practice, hysteroscopic adhesiolysis combined with physical barrier placement and cyclic estrogen therapy can achieve therapeutic benefit in patients with mild IUA. However, in moderate to severe endometrial injury, several limitations frequently emerge, including postoperative re-adhesion and inadequate endometrial responsiveness to hormonal stimulation. Recurrence rates in severe IUA have been reported to be as high as 62.5% [14].

Additional interventions have been explored, including systemic hormonal therapies such as human chorionic gonadotropin (hCG), gonadotropin-releasing hormone (GnRH), and growth hormone. Pharmacological agents to improve endometrial perfusion, such as aspirin and sildenafil, as well as biomimetic bioelectrical stimulation, have also been investigated [15, 16]. Nevertheless, robust clinical evidence demonstrating sustained improvements in endometrial function and reproductive outcomes remains insufficient.

**Fig. 1:**
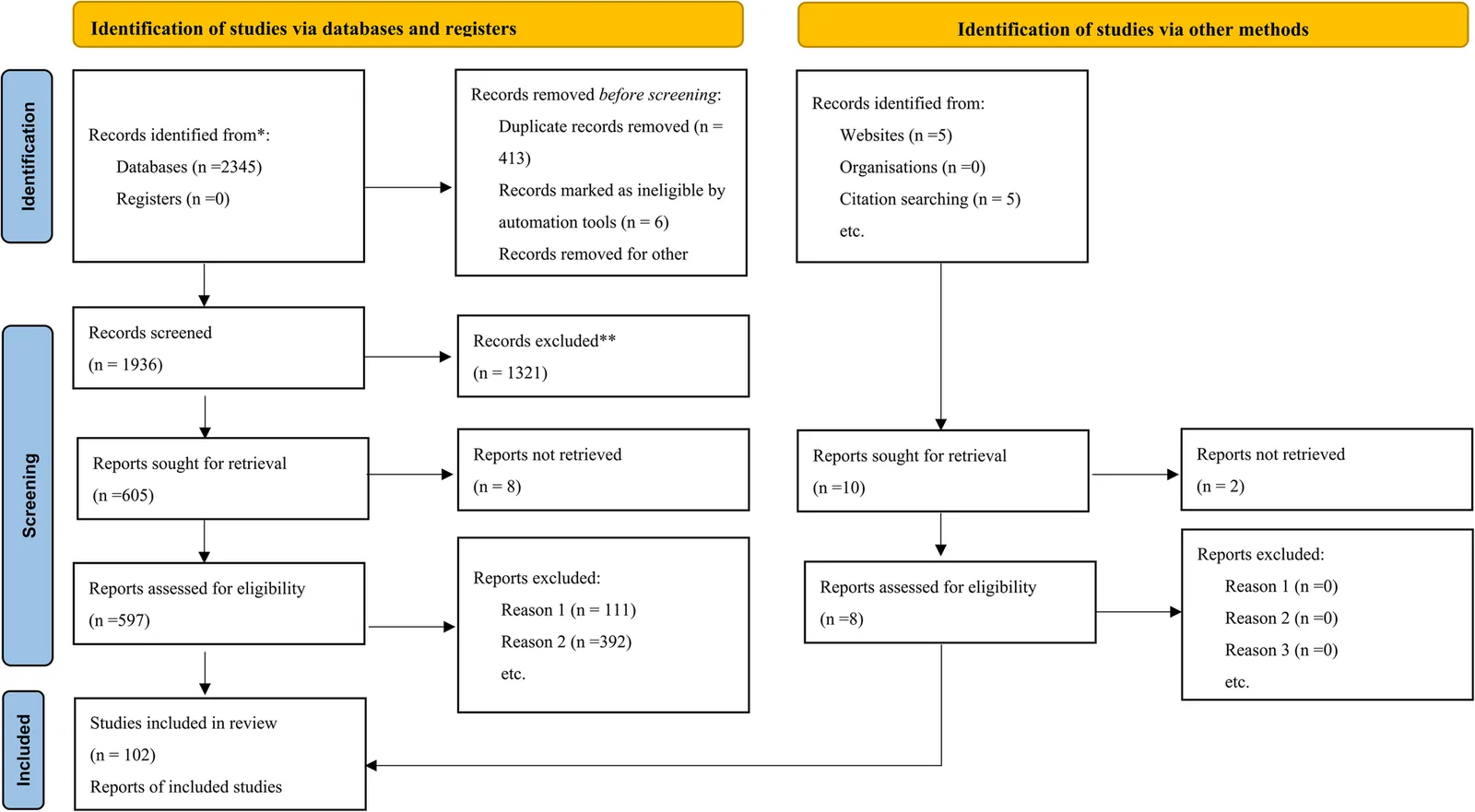
PRISMA 2020 guidelines. *Consider, if feasible to do so, reporting the number of records identified from each database or register searched (rather than the total number across all databases/registers). **If automation tools were used, indicate how many records were excluded by a human and how many were excluded by automation tools. Source: Page MJ, et al. BMJ 2021;372:n71. doi: 10.1136/bmj.n71. This work is licensed under CC BY 4.0. To view a copy of this license, visit https://creativecommons.org/licenses/by/4.0/

Accordingly, the development of innovative strategies to fundamentally promote endometrial regeneration and functional restoration has become a key research focus at the intersection of reproductive medicine and regenerative biology. The literature search and selection process for this review is summarized in Fig. 1.

This review aims to provide a systematic overview of advances in regenerative therapeutic strategies—specifically cell-based, cell-free, and bioengineering approaches—for endometrial repair. While acknowledging the importance of basic endometrial biology, this synthesis focuses on translational interventions designed to restore structural and functional competence.

## Cell Therapy

Cell therapy based on mesenchymal stem cells (MSCs) has emerged as the most extensively investigated approach in endometrial regeneration, supported by a comparatively substantial body of empirical evidence. These cells possess capacities for self-renewal and multilineage differentiation. In addition, they exert therapeutic effects through paracrine signaling, during which a range of growth factors is secreted. These bioactive factors facilitate the proliferation and differentiation of endometrial cells, while concurrently attenuating fibrotic processes and promoting angiogenesis. Through these coordinated mechanisms, the microenvironment of the damaged endometrium can be progressively optimized and functionally restored [17].

In studies on endometrial repair, mesenchymal stem cells derived from diverse tissues, including bone marrow, umbilical cord, endometrium, adipose tissue, and amniotic membrane, have been demonstrated to exhibit regenerative potential. Variations in surface marker profiles, differentiation capacity, and paracrine activity exist among MSCs from different sources, and these differences may influence their performance in specific repair contexts. Nevertheless, a growing body of evidence continues to support their translational and clinical relevance in endometrial regeneration [18]. A meta-analysis including 12 studies and 233 patients indicated that stem cell-based therapy was associated with improvements in menstrual recovery, pregnancy rate, live birth rate, and endometrial thickness in individuals with IUA. Adipose-derived and menstrual blood-derived stem cells showed a more pronounced capacity to increase endometrial thickness. Early-passage adipose-derived cells (P1) exhibit proliferative activity comparable to that of amniotic fluid-derived MSCs, while exerting stronger immunomodulatory effects than bone marrow-derived MSCs (BMMSCs) [19]. Regarding mechanistic insights and comparative efficacy, Gou et al. reported that the presence of specific markers in human endometrial stromal cells (EnSCs) enables more precise targeting of these cells. Their findings also suggest that BMMSCs may outperform EnSCs in uterine tissue regeneration [3]. Wang et al. [20] comparatively evaluated the reparative effects of mesenchymal stem cells from different tissue sources in a murine model. Transplantation of menstrual blood-derived EnSCs, bone marrow-derived MSCs, and umbilical cord-derived MSCs (UCMSCs) all led to increased endometrial thickness and enhanced tissue regeneration. No meaningful differences were observed between the menstrual blood-derived and bone marrow-derived groups in terms of endometrial thickness or integrin αVβ3 expression, whereas both outperformed the umbilical cord-derived group. Elevated levels of cytokeratin 18 and vimentin were also observed in the menstrual blood and bone marrow groups, indicating a more active regenerative response. Moreover, the expression pattern of vascular endothelial growth factor (VEGF) indicated that menstrual blood-derived EnSCs may have a greater capacity to promote angiogenesis and improve endometrial receptivity. Although these findings are encouraging, it must be noted that most existing studies have short follow-up periods, typically less than one year, and rely on surrogate endpoints such as endometrial thickness, while data on live birth rates remain scarce. Furthermore, because there is a lack of direct comparisons using standardized protocols across different sources of mesenchymal stem cells, it remains difficult to draw firm conclusions about their relative efficacy.

### Bone Marrow Mesenchymal Stem Cells (BMMSCs)

BMMSCs were an early cell type applied in endometrial regeneration research, yet their autologous acquisition requires invasive bone marrow aspiration, and their number and activity are influenced by donor age and health status.

Yu et al. demonstrated that, at the mechanistic level, crosstalk between BMMSCs and endothelial progenitor cells (EPCs) modulates the PI3K/Akt/Cox2 signaling axis. This interaction regulates apoptosis, proliferation, and differentiation, as well as the secretion of angiogenesis-related cytokines, thereby enhancing neovascularization and attenuating the progression of intrauterine adhesions [21]. In a prospective study, labeled BMMSCs were seeded onto an Interceed membrane and transplanted into the uterine cavity of an IUA rat model. Compared with the blank control, IUA model, Interceed-only, and BMMSCs-only groups, the combined Interceed-BMMSC treatment resulted in time-dependent increases in endometrial gland number, thickness, and gestational sac count. Concurrently, both mRNA and protein levels of LIF, integrin ανβ3, and VEGF in the endometrium were markedly elevated [22]. Recent studies have developed a dual-delivery platform incorporating BMMSCs and hyperoside. In this system, hyperoside embedded in the outer layer induces macrophage polarization toward the anti-inflammatory M2 phenotype, thereby modulating the early inflammatory microenvironment. The encapsulated BMMSCs exert paracrine effects that activate multiple signaling pathways, including Wnt/β-catenin, PI3K-Akt, and TGF-β. This activation facilitates the release of pro-angiogenic factors such as vascular endothelial growth factor and basic fibroblast growth factor, which in turn supports neovascularization and endothelial cell proliferation. Concurrently, the secretion of insulin-like growth factor-1 and hepatocyte growth factor contributes to the suppression of apoptosis and promotes the proliferation of residual endometrial cells. Remodeling of the extracellular matrix is also tightly controlled. Specifically, the balance between matrix metalloproteinases and their inhibitors is modulated, leading to the degradation of excessively deposited type I and type III collagen and the subsequent mitigation of fibrotic remodeling [23].

These in vitro findings support the clinical translation of stem cell-based therapies. In 2011, Nagori et al. reported the first clinical application in a patient with severe IUA. Autologous BMMSC transplantation combined with hormonal therapy increased her endometrial thickness from 3.6 mm to 8.0 mm, which led to a successful pregnancy [24]. This pioneering work has since spurred a growing number of clinical studies in the field. In 2016, investigators administered CD133⁺ BMMSCs via the uterine spiral artery to 11 patients with refractory IUA. Post-treatment assessment showed that endometrial thickness increased from 4.3 mm to 6.7 mm. Within three months, significant improvements were noted in vascular density, menstrual duration, and volume. However, these effects had returned to baseline by the six-month follow-up [25]. In 2020, a prospective study involving 25 patients (12 with IUA and 13 with TE) employed ultrasound-guided subendometrial infusion of BMMSCs in combination with estrogen therapy. Endometrial thickness increased from 3.3 ± 1.0 mm to 5.1 ± 1.9 mm after three months. Nevertheless, no statistically significant improvement was detected at 6 months, 9 months, or at the 5-year follow-up, and only three patients achieved pregnancy [26]. A pilot study in 2023 (*n* = 20) utilizing fractionated intrauterine transplantation of autologous bone marrow nucleated cells reported improvements in endometrial thickness in all but one participant, with half demonstrating uniform enhancement. Among the 12 patients who underwent embryo transfer, five achieved successful pregnancies, though only one resulted in a full-term delivery. The underlying mechanism is suggested to involve CD34⁺ cells releasing angiogenic factors, recruiting immune cells, and promoting vascular remodeling [27]. The randomized controlled trial by Zhu et al. showed that in patients with moderate to severe intrauterine adhesions, intrauterine delivery of an autologous BMMSC-loaded collagen scaffold via a Foley balloon catheter after adhesiolysis inhibited fibrosis and promoted endometrial regeneration through paracrine mechanisms. This treatment significantly increased the ongoing pregnancy rate (62.5% vs. 41.2%) and live birth rate (56.9% vs. 38.2%), with no serious adverse events except for a higher incidence of mild placenta accreta. These findings confirm that this therapy effectively improves pregnancy outcomes, particularly in patients with an AFS score of 8–10 [28]. More recently, a clinical study from China Medical University evaluated intrauterine infusion of autologous BMMSCs combined with hormone therapy. After three treatment cycles, both menstrual volume and endometrial thickness increased compared with baseline, a response likely associated with the paracrine activity of MSCs. However, the benefits were not sustained at the one- to two-year post-treatment follow-up. Two patients developed recurrent amenorrhea, and only one had a spontaneous pregnancy and delivery [29]. Collectively, these clinical studies reveal a recurring pattern: short-term improvements in endometrial thickness and menstrual patterns are often observed, but therapeutic gains are frequently not sustained beyond 6–12 months, and live birth rates remain disappointingly low. A critical limitation of this body of evidence is the lack of standardized protocols regarding cell dosage, administration route, and patient selection criteria, which precludes definitive conclusions about the true efficacy of BMMSCs. Moreover, no study to date has demonstrated that BMMSC therapy can selectively restore depleted regenerative subpopulations such as IGFBP3⁺ Stromal-1 cells or directly reactivate suppressed pathways like GZMA-PARD3, highlighting the gap between broad-spectrum repair and precision intervention. Accordingly, developing strategies to achieve sustained endometrial regeneration is a priority for future research.

### Umbilical Cord Mesenchymal Stem Cells (UCMSCs)

UCMSCs are primarily isolated from umbilical cord blood or Wharton’s jelly [30]. Owing to their abundance, robust proliferative capacity, low immunogenicity, and minimal risk of genetic instability, these cells have attracted considerable attention in studies of endometrial repair. Experimental evidence from both IUA and TE animal models indicates that UCMSCs can enhance endometrial regeneration and facilitate recovery of reproductive function.

The therapeutic actions of UCMSCs are mediated through multiple signaling pathways. In a rabbit model, these cells modulated the Th17/Treg immune balance via the NF-κB pathway, thereby restraining aberrant immune responses. This immunoregulatory effect was associated with an increase in endometrial gland number, elevated expression of estrogen receptors and Ki-67, and reduced fibrosis and TGF-β levels [31].

In a rhesus macaque model of intrauterine adhesion, a composite system consisting of UCMSCs and a self-crosslinking hyaluronic acid hydrogel was developed. This system served as a physical barrier to prevent re-adhesion while simultaneously enhancing endometrial regeneration via paracrine signaling and immune modulation [32]. For thin endometrium, encapsulation of UCMSCs in PF-127 has been reported to prolong cellular retention within the uterine cavity, which promotes the recovery of endometrial thickness and glandular density in rat models. Preconditioning with IL-1β further increases the release of pro-angiogenic factors from UCMSCs, thereby augmenting their reparative efficacy [33]. Reprogramming UCMSCs with inflammatory cytokines, including IL-1β, TNF-α, and IFN-γ, has also been shown to attenuate inflammation and fibrosis in murine IUA models. This effect is attributed to enhanced secretion of C1INH, suppression of the JAK-STAT pathway, and inhibition of CD301⁺ macrophage polarization toward a profibrotic phenotype [7]. This study evaluated the therapeutic effects of super-activated platelet lysate (sPL) and umbilical cord-derived mesenchymal stem cells in a rat model of thin endometrium. sPL is rich in growth factors such as PDGF and VEGF, which facilitate cell proliferation, angiogenesis, and fibrosis inhibition. UCMSCs act through paracrine mechanisms and differentiation into endometrial epithelial and stromal cells, synergizing with sPL to upregulate the expression of repair-associated proteins including cyclin D1, CD34, cytokeratin 18, and vimentin, while also restoring serum levels of PDGF-BB, TGF-β1, estradiol (E2), and follicle-stimulating hormone (FSH). The results demonstrated that treatment with sPL or UCMSCs significantly increased endometrial thickness (from approximately 181 μm to over 538 μm), elevated the number of glands and blood vessels, and improved tissue structural integrity. Furthermore, fibrosis and hormonal imbalances were reversed, indicating that sPL and UCMSCs effectively promote both morphological and functional regeneration of the thin endometrium [34]. Liu et al. demonstrated that miR-210-3p and miR-31-5p derived from hypoxic endometrial epithelial exosomes suppress UCMSC proliferation, migration, and differentiation toward endometrial epithelial cells by targeting SDF2 and FGF7, respectively, thereby negatively regulating the JAK2/STAT3 pathway. Inhibition of these miRNAs reversed the suppressive effects, enhanced cell functions, and upregulated epithelial markers such as CK19 and CD9. Conversely, overexpression of SDF2 or FGF7 activated the JAK2/STAT3 pathway and restored cell functionality. These findings highlight the critical role of the miR-210-3p/SDF2 and miR-31-5p/FGF7 axes in regulating UCMSC behavior [35].

To improve the engraftment efficiency and therapeutic efficacy of UCMSCs, various delivery and carrier systems have been investigated. Three-dimensional culture within alginate microcapsules has been shown to enhance cellular proliferation and antifibrotic activity. This effect is mediated by attenuation of the TGF-β1/Smad3 signaling pathway, leading to increased endometrial thickness, a higher number of glands, and improved fertility in murine models of intrauterine adhesion [36]. Combination strategies have also been explored. Co-administration with kaempferol appears to exert a synergistic regulatory effect on the JAK2/STAT3 pathway, thereby limiting epithelial-mesenchymal transition and reducing fibrotic progression [37]. In parallel, biomaterial-based scaffolds have shown promising supportive roles. Collagen scaffolds and decellularized human amniotic membranes have both been validated in rat models as effective matrices for UCMSC delivery, enabling structurally and functionally endometrial reconstruction [38, 39].

The therapeutic efficacy of UCMSCs is influenced by multiple variables, including the carrier platform, route of administration, and cell dosage. In a comparative study, Zhang et al. evaluated three delivery routes (intraperitoneal injection, local intrauterine administration, and tail vein injection) and three cell doses (0.5 × 10⁶, 1 × 10⁶, and 5 × 10⁶). The findings indicated that intraperitoneal delivery of 1 × 10⁶ cells achieved the most favorable outcomes for endometrial regeneration and attenuation of fibrotic changes [40].

Building upon prior animal experimental evidence [41], Zhang et al. conducted a prospective self-controlled study using a composite construct of UCMSCs and a biodegradable collagen scaffold. This strategy was applied to 18 patients with severe intrauterine adhesions or refractory thin endometrium, in combination with hormonal therapy. Post-treatment, endometrial thickness increased from 4.08 ± 0.26 mm to 5.87 ± 0.77 mm. The uterine artery systolic/diastolic (S/D) ratio decreased from 8.03 ± 2.31 to 6.53 ± 1.21, suggesting improved uterine perfusion. Microvessel density within the endometrium was elevated, accompanied by higher expression levels of Ki67, ERα, and PR, as well as an increased number of endometrial glands. Two patients subsequently achieved pregnancies and delivered live births [42].

While this study provides encouraging proof-of-concept data, several limitations warrant consideration. The self-controlled design lacks a concurrent control group, and the sample size is small (*n* = 18). The live birth rate (2/18, 11.1%) is modest, and the follow-up duration is not clearly specified, leaving questions about durability unanswered. A double-blind study at Xiangya Hospital evaluated UCMSCs loaded onto collagen scaffolds as a therapeutic intervention. A total of 24 patients completed follow-up. The cumulative clinical pregnancy rate in the treatment group reached 45.5%, whereas the control group showed a rate of 7.7%. In contrast, endometrial thickness was comparable between groups (6.5 ± 1.3 mm in the treatment group vs. 6.0 ± 1.2 mm in the control group), with no statistically significant difference. Notably, higher incidences of early miscarriage and placenta accreta were observed in the treatment cohort, and two cases of urticaria were reported during the study period. This dissociation between pregnancy rate and endometrial thickness is striking and suggests that mechanisms beyond simple structural expansion, such as immune modulation or paracrine effects on receptivity, may contribute to clinical outcomes. However, the increased rates of early miscarriage and placenta accreta raise safety concerns that require further investigation. The absence of long-term follow-up data on offspring health is another critical gap. Mechanistic analyses suggested that UCMSCs may exert their therapeutic effects predominantly through paracrine signaling and immunomodulation via cytokine-receptor interactions. These findings indicate a potential role of this approach in enhancing endometrial receptivity. Nevertheless, its long-term safety profile and ultimate influence on pregnancy outcomes require further rigorous evaluation [43].

### Endometrial Mesenchymal Stem Cells (EnSCs)

Endometrial stem cells (EnSCs) were conceptually proposed in 1978, and their successful isolation was later achieved by Chan and colleagues in 2004. Due to their robust differentiation capacity, relative accessibility, and favorable ethical profile, EnSCs are considered a promising adult stem cell source for regenerative applications [44, 45].

Hong and colleagues provided a comprehensive characterization of EnSC biology. They noted that EnSC function is closely associated with key markers (CD140b/CD146, N-cadherin, Musashi-1, LGR5, and side population cells) and signaling pathways, such as Notch and Wnt/β-catenin [46].

EnSCs are typically isolated from endometrial tissue or menstrual blood. Cells derived from menstrual blood, often termed MenSCs, are limited by low yield and contamination risk during collection and processing. In a porcine model of intrauterine adhesion induced by electrocautery injury, S100A8/A9 expression was markedly elevated. This upregulation promoted EnSC proliferation in a concentration-dependent manner. Mechanistically, S100A8/A9 interacts with the receptor for advanced glycation end products, leading to activation of the JAK2/STAT3 signaling pathway and driving the differentiation of EnSCs toward a myofibroblast phenotype, thereby facilitating fibrotic remodeling. Concurrently, expression of α-SMA and pro-inflammatory cytokines (e.g., IL-1β and IL-6) was increased. These findings further support a reparative role for MenSCs in endometrial injury [47]. Li et al. [13] demonstrated that EnSCs enhance the expression of the anti-inflammatory cytokine IL-10 while suppressing pro-inflammatory mediators such as IFN-γ and IL-2. Collagen deposition was reduced, and the expression of fibrosis-related markers, including Fn1 and Acta2, was downregulated. Endometrial repair is thought to involve multiple coordinated processes, including cellular homing, stimulation of cell proliferation, angiogenesis, and glandular regeneration, although the in vivo mechanisms remain to be further elucidated. Furthermore, when MenSCs were induced to differentiate into decidualized stromal cells (DSCs), their VEGF-A secretion increased approximately twofold. This was associated with enhanced pro-angiogenic capacity and superior fertility restoration compared with undifferentiated cells [48]. An unresolved question is whether the reparative effects of MenSCs are primarily mediated by direct differentiation into endometrial cell lineages or by paracrine signaling. Most evidence points toward a paracrine-dominant mechanism, given the low engraftment rates typically observed. This distinction has important implications for safety and scalability, as paracrine-based effects might be recapitulated by cell-free approaches with lower risk profiles.

Advances in animal models have provided a conceptual and experimental basis for subsequent clinical translation. In one clinical study, fifteen patients received ultrasound-guided multipoint injections of MenSCs into the unilateral ovarian cortex. Improvements in ovarian reserve parameters were observed, and spontaneous pregnancies occurred in several cases. The proposed mechanism involves the secretion of paracrine factors such as VEGF and IGF-1. These factors may enhance local microcirculation, attenuate follicular apoptosis, facilitate residual follicle activation, and modulate the ovarian immune microenvironment, thereby limiting autoimmune-mediated damage. These findings should be interpreted with caution, as the study lacked a control group and had methodological limitations [49]. Peng et al. administered autologous EnSCs via intrauterine infusion in combination with sodium hyaluronate gel, estrogen therapy, and aspirin in fifteen patients with endometrial injury. The overall pregnancy rate reached 60.0% (9/15), and the live birth rate was 53.3% (8/15). RNA sequencing analysis suggested that the therapeutic effect may be associated with restored cytokine signaling homeostasis and rebalanced inflammatory pathways involved in endometrial repair [50]. While these pregnancy and live birth rates appear promising, the small sample size (*n* = 15), absence of a control group, and concurrent use of multiple co-interventions (sodium hyaluronate gel, estrogen, aspirin) make it difficult to attribute the observed effects specifically to EnSCs. Furthermore, the lack of long-term follow-up data on offspring health and maternal outcomes limits the ability to assess safety comprehensively.

### Mesenchymal Stem Cells from Other Sources

Adipose-derived mesenchymal stem cells (ADSCs) have emerged as a feasible cell source owing to their accessibility and regenerative potential. In a pilot clinical study (*n* = 5), autologous ADSCs were harvested via liposuction and administered through cervical infusion in combination with estrogen therapy. Endometrial thickness increased from 3.0 ± 1.0 mm to 6.9 ± 2.9 mm, accompanied by alleviation of menstrual symptoms. One patient achieved pregnancy but miscarried at nine weeks of gestation. These preliminary outcomes suggest therapeutic potential; however, both the durability of efficacy and long-term safety require validation in larger, well-controlled cohorts [51]. The extremely small sample size (*n* = 5) and lack of a control group in this pilot study severely limit the generalizability of the findings, and the single miscarriage without live birth reported raises questions about functional efficacy. Combination approaches have also been investigated to enhance cellular performance. Co-administration of ADSCs with platelet-rich plasma (PRP) activates the Akt/ERK signaling cascade. This activation supports cell survival, proliferation, and migration without impairing adipogenic differentiation. ADSCs promote regeneration of uterine and ovarian tissues through pro-angiogenic, antifibrotic, and paracrine activities. PRP provides an additional source of growth factors, thereby potentiating these biological actions through complementary mechanisms. Evidence from preclinical models and early clinical observations indicates that this combinatorial strategy may contribute to the restoration of endometrial receptivity and partial recovery of ovarian function [52].

Amnion-derived mesenchymal stem cells (AMMSCs) represent another promising cell source for endometrial repair. Huang et al. [53] demonstrated that co-culturing human AMMSCs with TGF-β1-induced endometrial stromal cells from an IUA model attenuated profibrotic signaling, particularly the TGF-β/Smad pathway. This modulation was accompanied by reduced expression of fibronectin and type I collagen, together with enhanced matrix metalloproteinase (MMP) activity, indicating enhanced extracellular matrix remodeling. In parallel, AMMSCs promoted angiogenesis by secreting vascular endothelial growth factor and basic fibroblast growth factor. Anti-inflammatory and immunoregulatory effects were also observed, mediated in part by the release of prostaglandin E2 and interleukin-10. Collectively, the evidence suggests that AMMSCs exert reparative effects primarily through paracrine signaling rather than direct cellular replacement. To date, most of the data on using amniotic membrane derived mesenchymal stem cells, or AMMSCs, for endometrial repair have come from in vitro or animal studies. Therefore, the clinical translation of AMMSCs is still in its early stages. Before these cells can be considered for clinical use, a number of major obstacles must be addressed, including how to achieve large scale production, how to ensure quality control, and how to obtain regulatory approval.

### Challenges and Perspectives: From Broad-Spectrum Repair to Precision Intervention

Although MSC transplantation has shown considerable therapeutic promise for endometrial regeneration, several critical barriers hinder its clinical translation. Most available studies focus on nonspecific, broad-spectrum repair, whereas targeted interventions directed at defined pathological subpopulations or signaling axes are largely lacking. For instance, reduced IGFBP3⁺ Stromal-1 cells and suppression of the GZMA-PARD3 signaling axis, as described in patients with thin endometrium, are now regarded as key pathogenic features. However, convincing evidence is lacking that MSCs from bone marrow, umbilical cord, or menstrual blood can selectively restore the abundance or function of this stromal subset. Likewise, targeted approaches, such as exosome-based delivery of GZMA or PARD3 mimetics for pathway-specific repair, have not been systematically reported. This disconnect between mechanistic discovery and therapeutic development is a major obstacle, preventing regenerative medicine from progressing beyond descriptive observations toward causal, precision-based interventions. To fill this research gap, future studies should use advanced technological platforms such as single cell sequencing, spatial transcriptomics, and organoids. These tools can help identify stem cell subsets or engineered exosomes that are capable of preferentially homing to the injured endometrium and reversing key molecular abnormalities. Without such targeted strategies, the field will struggle to move beyond its current reliance on empirical, broad spectrum repair. It will also find it difficult to reach a stage of mechanism guided precision intervention.

The quality of existing clinical evidence varies considerably, and data on long-term safety and durability remain limited. Several small-scale studies have reported short-term increases in endometrial thickness and improvements in menstrual volume following stem cell therapy. However, effects on live birth rates have been inconsistent, and follow-up in most studies has not exceeded one year (Table 1). In some cohorts, therapeutic benefits diminished within six months, with certain patients returning to baseline status or even experiencing recurrent amenorrhea. These findings suggest that a single-administration strategy may be insufficient to remodel an established chronic fibrotic microenvironment. A key issue is that most current studies rely on surrogate endpoints, such as endometrial thickness or gland density, which have limited value for predicting live birth. Therefore, it is recommended that regulatory agencies and relevant professional societies promote the use of patient centered endpoints, including cumulative live birth rate, miscarriage rate, and pregnancy complications, as the main benchmarks for assessing treatment effectiveness. Without this shift, the clinical relevance of any reported benefits will be difficult to confirm. Future investigations should prioritize multicenter randomized controlled trials with live birth rate as the primary endpoint and follow-up extending beyond two years. In parallel, standardized release criteria for cellular potency need to be established to ensure product consistency and reproducibility across studies.

**Table 1:** Comparative profile of bone marrow-, umbilical cord-, and endometrial-derived MSCs in endometrial repair

Feature	BMMSCs	UCMSCs	EnSCs
Key advantages	Longest research history; most extensive evidence base; robust tissue regeneration potential	Widely available; ethically non‑controversial harvest; high proliferative capacity; low immunogenicity; low mutation risk	High endometrial tropism; easily accessible; favorable ethical profile; promising potential for improving endometrial receptivity [40, 41]
Key limitations	Invasive harvest; donor age/health affecting cell quality; uncertain long‑term clinical efficacy	Optimal delivery route/dose yet to be defined; long‑term safety and offspring effects require further validation	Limited yield and contamination risk in menstrual blood‑derived cells; in vivo mechanisms insufficiently characterized
Core mechanisms	Paracrine secretion of growth factors promoting angiogenesis and cell proliferation; activation of repair pathways (Wnt/β‑catenin, PI3K/Akt); anti‑fibrotic via MMPs/TIMPs balance, degrading excessive collagen [20–22]	Immunomodulation (NF‑κB, JAK‑STAT); pro‑angiogenic (VEGF, enhanced by preconditioning); anti‑fibrotic (inhibition of TGF‑β1/Smad3) [31–33]	Homing and differentiation; paracrine/angiogenic (e.g., VEGF); immunomodulation and anti‑fibrosis via upregulation of anti‑inflammatory cytokines and downregulation of pro‑inflammatory cytokines/fibrosis markers [12, 43, 44]
Preclinical evidence	Improved endometrial thickness, gland count, and pregnancy outcomes in animal models [20–22]	Enhanced endometrial repair, increased glands, reduced fibrosis, restored fertility [31–33]	Promoted endometrial regeneration, increased glands/thickness, inhibited fibrosis/inflammation [12, 43, 44]
Clinical evidence	Short‑term increases in endometrial thickness and menstrual improvement; occasional successful pregnancies [23–26]	Increased endometrial thickness and improved uterine blood flow; reported live births but pregnancy rate requires improvement [37–39]	Improved endometrial parameters and increased pregnancy/live birth rates; partial restoration of ovarian function [45, 46]
Level of evidence	Moderate: Prospective cohort studies; 5‑year follow‑up reported; lack of RCTs; small samples; high heterogeneity	Moderate: RCT available (Xiangya double‑blind trial) and self‑controlled studies; long‑term data insufficient	Low to moderate: Primarily before‑after studies; small samples; mechanistic studies outnumber efficacy validation

Regarding delivery approaches, intrauterine infusion often results in limited cellular retention and reduced viability. Arterial administration shows constrained homing efficiency, while subendometrial injection may lack precise localization within the injured tissue. To address these limitations, integrating MSCs with biocompatible carriers has emerged as a key strategy to improve engraftment and sustain therapeutic activity. Comparative analyses of different MSC sources indicate distinct biological advantages. ADSCs are readily accessible and available in abundant quantities. BMMSCs may exhibit superior pro-angiogenic capacity. UCMSCs are characterized by low immunogenicity, supporting their allogeneic application. EnSCs, in contrast, appear to have unique potential for enhancing endometrial receptivity. Regulatory and ethical considerations also warrant careful attention. Transparent reporting, rigorous study design, and comprehensive informed consent are essential to ensure both scientific integrity and patient safety in the clinical translation of stem cell-based therapies.

While MSCs universally improve endometrial thickness, the underlying mechanisms differ substantially by tissue source, explaining the heterogeneity in clinical outcomes. BMMSCs exert their effects primarily through the PI3K/Akt/Cox2 axis, which optimizes the interaction with endothelial progenitor cells to enhance neovascularization [21]. In contrast, UCMSCs modulate the Th17/Treg balance via NF-κB providing superior immunomodulation but potentially carrying a higher risk of aberrant placental adherence if differentiation is not strictly controlled [31, 43]. ADSCs, conversely, demonstrate a unique advantage in paracrine secretion of IGF-1 and HGF, which are critical for suppressing residual stromal cell apoptosis. Understanding these mechanistic nuances is essential for selecting the appropriate cell type for specific patient subtypes (e.g., pure atrophy vs. severe fibrosis).

## Cell-Free Therapy

Genomic and phenotypic instability inherent in MSCs raises safety concerns that constrain their clinical translation, particularly with respect to tumorigenic potential, immunogenicity, and source-related variability. In light of these considerations, research has increasingly focused on cell-free therapeutic modalities, such as platelet-rich plasma, extracellular vesicles (EVs), and microRNAs (miRNAs).

These approaches act via bioactive factors secreted by stem cells or specific tissues, thereby avoiding the risks associated with live cell transplantation. They generally offer greater biological stability, reduced immunogenicity, and improved accessibility. Their therapeutic actions involve the modulation of miRNA profiles, cytokine networks, and multiple signaling pathways. Through these coordinated mechanisms, endometrial fibrosis and inflammation may be attenuated, angiogenesis facilitated, and endometrial receptivity and function restored. Despite these advantages, several key issues remain unresolved. The precise molecular mechanisms have not been fully delineated. The optimal therapeutic window is still uncertain, and efficacy in different patient subgroups requires further investigation. Robust evidence from multicenter randomized controlled trials with extended follow-up will be essential to establish their long-term safety and clinical effectiveness.

### Platelet-Rich Plasma (PRP)

Platelet-rich plasma (PRP) represents the most commonly applied cell-free therapeutic modality in current clinical practice. It is a biological preparation derived from autologous peripheral venous blood through centrifugation and is enriched in platelets as well as a broad spectrum of cytokines and growth factors. PRP exhibits immunomodulatory properties and may enhance endometrial receptivity, in part through the suppression of pro-inflammatory mediators, including IL-6 and IL-1β [54].

Mechanistic studies have further elucidated the context-dependent actions of PRP. In an in vitro study, Kuroda et al. [55] reported that PRP exerts differential effects on undifferentiated and decidualized endometrial stromal cells (EnSCs). In undifferentiated cells, PRP enhances cellular proliferation, tissue repair, and pro-inflammatory activity, largely mediated by growth factors such as TGF-β and PDGF. In contrast, in decidualized cells, PRP exhibits an anti-inflammatory profile. This effect is associated with PTEN upregulation and subsequent inhibition of the PI3K/AKT signaling pathway, which may enhance intrauterine immune tolerance and create a more receptive endometrial environment for embryo implantation. Nevertheless, the study was constrained by a limited sample size and the absence of dose-response evaluation for PRP. Complementary evidence from an animal model of intrauterine adhesion demonstrated that PRP promotes endometrial regeneration through multiple molecular pathways. Mao et al. [56] showed that PRP upregulates melanotransferrin expression and activates pathways related to retinol metabolism and ECM-receptor interaction, thereby facilitating tissue reconstruction. In addition, PRP attenuates cell death through the modulation of ferroptosis, autophagy, and pyroptosis, suggesting a multifaceted role in maintaining endometrial cellular homeostasis.

Animal studies have demonstrated that combining MenSCs with PRP enhances endometrial repair more effectively than either treatment alone. Among the examined markers, the cell densities of CK18, vimentin, and integrin αVβ3 exhibited the most pronounced increases in the combination group [57].

Clinical outcomes have been evaluated in several studies with encouraging results. In a small-scale study of nine patients with TE, all participants exhibited increased endometrial thickness following PRP infusion, with increments ranging from 0.7 mm to 2.2 mm. Clinical pregnancy was achieved in all cases, although the limited sample size and short follow-up period precluded assessment of live birth rates [58]. In a separate study including 200 TE patients, intrauterine PRP injection combined with estrogen-progestogen therapy resulted in greater endometrial thickness in the treatment group compared with controls (7.7 ± 1.9 mm vs. 6.1 ± 1.2 mm). The intervention also reduced the rate of cycle cancellation due to endometrial inadequacy (34.0% vs. 75.0%) and led to spontaneous pregnancies in three patients [59]. A large retrospective study of 220 patients indicated that intrauterine infusion of autologous PRP significantly improved uterine hemodynamic parameters, including the pulsatility and resistance indices of the bilateral uterine, arcuate, and spiral arteries. The intervention was associated with higher live birth, clinical pregnancy, and ongoing pregnancy rates. Potential mechanisms include the upregulation of endometrial receptivity markers (e.g., integrin αvβ3), activation of signaling pathways (e.g., PI3K/Akt), and modulation of the endometrial microenvironment. This modulation is achieved through growth factor release, stimulation of endogenous stem cells, immune regulation (notably promoting M2 macrophage polarization), and coordinated enhancement of angiogenesis. These combined effects contribute to improved endometrial thickness and uterine perfusion, thereby facilitating favorable pregnancy outcomes. No severe adverse events were reported, although the short follow-up period limits the availability of long-term data [60]. A recent meta-analysis compared multiple therapeutic approaches for TE, including oral aspirin, Dingkundan, intrauterine infusion of PRP, intrauterine administration of granulocyte-macrophage colony-stimulating factor (GM-CSF), intramuscular injection of recombinant human growth hormone, and neuromuscular electrical stimulation. In terms of endometrial thickness, GM-CSF showed the greatest efficacy, followed by aspirin, PRP, Dingkundan, neuromuscular electrical stimulation, and recombinant human growth hormone, with the control group ranking lowest. For clinical pregnancy rates, PRP demonstrated the highest efficacy, followed by aspirin, Dingkundan, GM-CSF, recombinant human growth hormone, neuromuscular electrical stimulation, and the control group. These findings indicate that PRP provides a combined advantage in both endometrial expansion and pregnancy outcomes [61].

PRP has also been investigated for its potential to enhance ovarian reserve. A systematic review reported that PRP is rich in multiple growth factors (e.g., PDGF, VEGF, TGF-β), clotting factors, and differentiation-promoting molecules, which collectively promote angiogenesis, immune regulation, and tissue repair. Meta-analytic evidence suggests that intra-ovarian PRP administration improves levels of follicle-stimulating hormone (FSH) and anti-Müllerian hormone (AMH), potentially activating ovarian function within one month. The observed improvements were maintained for at least three months, although the precise duration of effect remains to be established [62].

Clinical studies investigating PRP exhibit substantial heterogeneity, primarily attributable to the lack of standardized preparation protocols. The most prominent barrier to PRP clinical translation is the lack of unified preparation protocols, leading to the “same-name heterogeneity” phenomenon. A synthesis of the 14 PRP studies included in a review found that key parameters varied widely: centrifugal protocols ranged from single-spin (1500 rpm, 10 min) to double-spin (first 3000 rpm, 15 min; second 2000 rpm, 10 min), platelet enrichment multiples spanned 1.5–5.2× baseline, and leukocyte content differed by more than 8-fold between leukocyte-rich and leukocyte-poor PRP. Correspondingly, the concentration of core growth factors (VEGF, PDGF, TGF-β) in the final product varied by 4.3–11.7× across studies [63].

Yang et al. compiled the key parameters of PRP preparation from seven studies and revealed considerable heterogeneity. Regarding centrifugation protocols, the number of steps ranged from one to two. Centrifugal force was expressed either as relative centrifugal force in g (200 g, 1017 g, 3600 g) or as non-standardized rotational speed in rpm (1200 rpm, 3300 rpm), making direct comparisons of separation efficiency impossible. Centrifugation time also varied widely, from 3 to 15 min. Moreover, blood collection volume and anticoagulant ratios differed substantially: the volume of blood drawn ranged from 8.5 mL to 18 mL, and the amount of anticoagulant (ACD-A) used was between 1.5 mL and 2.5 mL. Infusion volumes were equally inconsistent, from as low as 0.5 mL up to 4 mL. These wide variations in parameters consequently lead to marked fluctuations in platelet enrichment, leukocyte residue, and growth factor concentrations. This lack of standardization not only undermines the reliability of cross-study comparisons but also makes it difficult for evidence-based methods, such as meta-analysis, to draw conclusions that can truly inform clinical practice [64].

According to the SIMCRI 2026 Expert Consensus on Regenerative Medicine, autologous platelet-rich plasma (PRP) should be prepared using a double centrifugation method within a closed sterile system. The use of EDTA is prohibited; sodium citrate or ACD-A is preferred as the anticoagulant, with an anticoagulation ratio of approximately 9:1. The final product shall have a platelet concentration of 1.0 × 10⁶/µL ± 20%, an enrichment factor of 4–5 times baseline, and a platelet recovery rate ≥ 50%. The clinically effective total platelet dose shall be no less than 3.2 × 10⁹. Leukocyte-poor PRP (P-PRP) is routinely recommended, and red blood cells should be removed as thoroughly as possible. The preparation shall be used immediately after fresh preparation; platelet lysate may be stored at − 80 °C for no longer than 9 months. However, the consensus states that its coverage of medical fields is limited, and further research is needed for its application in the reproductive field [65].

Another critical limitation is the frequent reliance on surrogate endpoints, particularly endometrial thickness, as the primary outcome measure. While endometrial thickness is easily quantifiable and shows a moderate association with embryo implantation, its sensitivity and specificity in predicting pregnancy success are limited. Several studies have demonstrated that PRP can increase endometrial thickness without necessarily improving live birth rates, and in some cases, improvements in thickness have been achieved without corresponding gains in pregnancy outcomes. This dissociation suggests that endometrial thickness may be an inadequate surrogate for true functional recovery. In contrast, patient-centered hard endpoints such as live birth rate are rarely reported in existing studies. Most studies have follow-up periods that only cover a single embryo transfer cycle or the early postpartum period, lacking systematic assessment of long-term offspring health and maternal outcomes. Therefore, when interpreting conclusions about the ability of PRP to improve pregnancy outcomes, caution should be exercised based on sample size, the presence or absence of control groups, and potential biases in endpoint selection. We recommend that future clinical trials adopt live birth rate as the primary endpoint and extend follow-up to at least 12 to 24 months postpartum in order to comprehensively evaluate the long-term efficacy and safety of the treatment.

Regarding the administration route, a meta-analysis demonstrated that subendometrial PRP injection yields higher clinical pregnancy and live birth rates and a lower miscarriage rate compared with intrauterine infusion [66]. In a study by Feng et al. of 100 patients with thin endometrium, the efficacy of single versus double intrauterine PRP infusions was compared. Double infusions administered on days 11 and 13 resulted in greater improvements in endometrial receptivity and clinical pregnancy outcomes than a single infusion on day 11. However, this study excluded patients with recurrent implantation failure or recurrent miscarriage; therefore, its findings warrant validation through larger-scale trials [67].

### Extracellular Vesicles (EVs)

EVs are nanoscale, lipid-bilayer-enclosed structures secreted by cells. Carrying proteins, nucleic acids, and other bioactive molecules, they function as critical mediators of intercellular communication. Based on their biogenesis and size, EVs are generally classified into three main types: exosomes, microvesicles, and apoptotic bodies. They play important roles in tissue repair, antifibrotic processes, and immune regulation [18]. Although large-scale clinical applications have not yet been realized, EVs are considered a promising therapeutic modality due to their high biocompatibility, low immunogenicity, and ease of storage.

MSC-derived EVs have been shown to promote endometrial repair through multiple mechanisms. In a rat model of intrauterine adhesion, Mansouri-Kivaj et al. [68] reported that both allogeneic MSCs and their EV20K preparations downregulated the pro-inflammatory cytokine TNF-α, upregulated the anti-inflammatory cytokine IL-10, and increased the expression of molecules associated with endometrial receptivity, such as VEGF and LIF. These effects resulted in inhibition of fibrosis and inflammation, alongside promotion of cellular proliferation. Proteins contained within exosomes, including heat shock proteins and integrins, modulate inflammatory responses, enhance angiogenesis, and support cell adhesion. Lipid components contribute to target cell recognition and improve vesicle stability and delivery efficiency [2]. Exosomes derived from UCMSCs preconditioned with transforming growth factor-β1 (TGF-β1) exhibited greater inhibition of the TGF-β1/Smad2/3 signaling pathway and fibrosis than those from naïve UCMSCs. This enhanced anti-fibrotic effect was associated with improved endometrial thickness, glandular density, receptivity, and pregnancy outcomes [69].

Huo et al. found that exosomes derived from human umbilical cord mesenchymal stem cells (hUMSC-Exo) deliver lncRNA IGF2R, which sponges miR-143-5p, leading to upregulation of AQP8. This suppresses the transformation of endometrial stromal cells into myofibroblasts and reduces the expression of fibrotic molecules such as collagens and α-SMA. Animal experiments showed that this treatment increased endometrial thickness and gland number, restored ER/PR/HOXA10 expression, and improved litter size. Knockdown of AQP8 or depletion of IGF2R in exosomes abolished the anti-fibrotic effects, confirming the key regulatory role of the lncRNA IGF2R/miR-143-5p/AQP8 axis [70].

The reparative effects of EVs are often mediated by key molecular pathways involving specific miRNAs that they carry. In a TGF-β1-induced fibrotic environment, MSC-derived exosomes showed marked upregulation of miR-145-5p, which may mediate tissue repair through a P62-dependent autophagy pathway [71]. Cao et al. [72] demonstrated that miR-27b-3p, miR-145-5p, and miR-16-5p carried by human embryonic stem cell-derived exosomes directly target Smad2, inhibiting its phosphorylation and thereby modulating the TGF-β/Smad signaling pathway to counteract fibrosis. Additionally, exosomes from hypoxia-preconditioned BMMSCs delivered miR-424-5p, which suppressed DLL4 and regulated the DLL4/Notch pathway. This led to the promotion of angiogenic factor expression and enhanced endometrial repair [73]. Liang et al. [74] found that placental MSC-derived exosomes deliver miR-143 to inhibit MyD88, thereby modulating the NF-κB pathway, reducing inflammation and fibrosis, and facilitating endometrial regeneration.

Zhang et al. reported that miR-137-3p is markedly upregulated in exosomes derived from endometrial epithelial cells under hypoxic injury. Once internalized by human umbilical cord mesenchymal stem cells (hucMSCs), this miRNA directly targets the 3′-UTR of UBE3C and suppresses its expression, leading to activation of the STAT3 signaling pathway (as indicated by elevated p-STAT3 levels). Consequently, miR-137-3p enhances hucMSC migration and induces their differentiation into endometrial epithelial cells, manifested by increased expression of the epithelial markers CK19 and CD9 and decreased expression of the stromal markers vimentin and CD13 [75]. Peng and colleagues developed a snail mucus-enhanced adhesive hydrogel loaded with folic acid-modified extracellular vesicles and STAT1 siRNA. Following intrauterine injection, the hydrogel firmly adheres to the injured site and delivers STAT1-siRNA in a manner that targets M1 macrophages. This inhibits STAT1 phosphorylation, thereby reducing M1 polarization, the release of pro-inflammatory cytokines, myofibroblast transformation, and collagen deposition. In animal experiments, the hydrogel significantly alleviated intrauterine adhesions, promoted endometrial regeneration, and markedly improved pregnancy rates and fetal numbers, ultimately restoring fertility [76]. Collectively, these findings indicate that exosomes from diverse cellular origins can deliver specific miRNAs to modulate multiple critical signaling pathways, representing a novel strategy for endometrial repair.

A recent meta-analysis of preclinical studies further confirmed that stem cell-derived EVs effectively increase endometrial thickness and glandular density while reducing fibrosis. These effects were mediated by the reversal of fibrosis-associated markers (e.g., TGF-β1, α-SMA, and Col-1) and the activation of proliferative and angiogenic pathways (e.g., VEGF and Ki67). The analysis also revealed that EVs from distinct sources, including adipose tissue, bone marrow, menstrual blood, and umbilical cord, exhibited statistically significant differences in their effects on glandular number, fibrotic area, and endometrial thickness [77]. Although EVs derived from bone marrow, umbilical cord, adipose tissue, placenta, and menstrual blood have all been shown to alleviate endometrial fibrosis and inflammation and to promote tissue regeneration [78], direct comparative studies among these sources remain scarce, and their long-term safety has yet to be comprehensively evaluated. A critical unresolved issue is whether the observed reparative effects of extracellular vesicles are primarily mediated by their miRNA cargo, by surface receptor interactions, or by the transfer of functional proteins. Understanding these mechanisms is essential for the rational design of extracellular vesicle-based therapies and for quality control. Furthermore, most of the current evidence evaluating extracellular vesicles for endometrial repair in humans comes from preclinical models. The translational pathway for extracellular vesicles faces major challenges, including large-scale production, standardization of isolation methods, stability during storage, and demonstration of safety in human subjects.

### MicroRNAs (miRNAs)

Specific miRNAs play pivotal roles in regulating endometrial fibrosis, inflammation, and angiogenesis. Consequently, the delivery of synthetic miRNA mimics with defined functions has emerged as a potential therapeutic approach for endometrial injury. However, this field remains largely at the stage of mechanistic exploration and optimization of delivery systems.

Studies have demonstrated that miR-455-5p, when overexpressed in UCMSCs, targets and suppresses SOCS3, thereby activating the JAK/STAT3 signaling pathway and promoting endometrial repair [79]. In a murine model of intrauterine adhesion, miR-10a-loaded liposomes facilitated angiogenesis by modulating macrophage polarization, thereby contributing to the restoration of uterine function [80]. Additionally, the long non-coding RNA MALAT1 enhances the reparative capacity of MenSCs by competitively binding miR-7-5p, thereby relieving its inhibitory effect on the target gene TCF4 and activating the Wnt signaling pathway. This mechanism improves endometrial thickness and supports favorable fertility outcomes [81].

Despite these encouraging mechanistic insights, the translation of miRNA-based therapies still faces substantial challenges. These challenges include off-target effects, the immunogenicity of synthetic miRNA mimics, the need for efficient and safe delivery systems that can specifically target the endometrium, and the lack of reliable animal models that faithfully recapitulate human endometrial pathophysiology. Furthermore, the field is still at an early preclinical stage.

We compared PRP and EVs in terms of their core components, primary mechanisms, research progress, major advantages, and major limitations (Table 2).

**Table 2:** Comparative profile of PRP and EVs in endometrial repair

Feature	Platelet-Rich Plasma (PRP)	Extracellular Vesicles (EVs)
Core components	Autologous blood derivative enriched with VEGF, TGF‑β, PDGF, and other growth factors [50]	Proteins, lipids, nucleic acids, and other bioactive molecules
Primary mechanisms	Immunomodulation: downregulation of IL‑6, IL‑1β [50]; Tissue repair: growth factor supply, activation of endogenous stem cells, angiogenesis, cell proliferation; Receptivity enhancement: upregulation of integrin αvβ3, activation of PI3K/Akt [51, 52, 56]	Intercellular communication: delivery of functional cargo to target cells [2]; Multi‑pathway regulation: specific miRNAs [64, 65] targeting TGF‑β/Smad [63], DLL4/Notch [66], NF‑κB [67]; Synergistic inhibition of fibrosis/inflammation and pro‑angiogenic effects
Research progress	Most widely used cell‑free therapy in clinical settings; multiple clinical studies (n=9–220) demonstrated improvements in ovarian function, endometrial thickness, uterine blood flow, and pregnancy rates [55–58]	Abundant preclinical evidence; high translational potential; therapeutic efficacy varies by cell source [68, 69]
Major advantages	Autologous origin → high safety profile; low immunogenicity; relatively simple preparation; substantial clinical data available	High biocompatibility and low immunogenicity; circumvents tumorigenic and other risks associated with live cell transplantation; diverse mechanisms of action
Major limitations	Preparation heterogeneity: Lack of standardized centrifugation protocols, activation methods, and quality control → substantial batch‑to‑batch variability, poor comparability across studies; Surrogate endpoint reliance: Most studies use endometrial thickness as primary endpoint, with scarce live birth rate data and short follow‑up; Dose‑response relationship undefined	Standardization gap: EV isolation methods (ultracentrifugation, tangential flow, precipitation) yield variable purity and quantity; no consensus on particle size/marker identification; Poor in vivo homing: short intrauterine retention; Long‑term safety unknown: Nucleic acid cargo may carry pro‑tumorigenic or epigenetic risks

### Challenges and Perspectives

Cell-free therapies, such as PRP and EVs, have demonstrated significant therapeutic potential; however, their clinical translation faces several challenges.

Pharmacokinetics and delivery systems require optimization, as EVs exhibit a relatively short in vivo half-life, necessitating strategies to prolong their retention at target sites. Optimal administration routes, dosing regimens, and long-term safety data remain poorly characterized. Heterogeneity and lack of standardization further complicate clinical translation. For EVs, biological properties depend on cellular origin, and isolation and purification methods are not yet standardized [18]. For PRP, key parameters—including platelet concentration and leukocyte content—vary with procedural differences, leading to inconsistent efficacy.

Universally accepted reference standards for PRP and EV preparations are still lacking. This remains a major obstacle to comparing results across studies and conducting meaningful meta-analyses. Establishing such standards should therefore be made a high priority in the field.

Evidence quality and mechanistic understanding are limited, with most data derived from animal models and high-quality clinical studies still lacking. Long-term safety and efficacy assessments are also absent, as follow-up periods in current studies are typically brief and fail to systematically evaluate delayed outcomes or potential risks.

Although cell-free therapies offer safety advantages over live cell-based approaches, their mechanisms of action often remain incompletely understood. For example, it is still unclear whether PRP acts primarily through growth factor-mediated proliferation, immune modulation, or activation of endogenous stem cells. Similarly, for EVs, it is not known whether the therapeutic effect requires continuous signaling from live cells or can be achieved with a single bolus of vesicles. Addressing these mechanistic questions will be essential for optimizing dosing regimens and predicting patient responses.

Future directions for advancing clinical translation of PRP and EVs include establishing international consensus and standardized protocols for their preparation and quality control. The development of targeted delivery systems could enhance retention and specificity at lesion sites. Exploring the synergistic effects of combined therapies, such as integration with biomaterials, may further enhance therapeutic outcomes. A deeper understanding of the key mechanisms underlying tissue repair will enable the development of personalized therapeutic strategies. Ultimately, multicenter trials with large sample sizes and extended follow-up periods are required to confirm both safety and efficacy.

## Bioengineering Strategies

To address the limitations of stem cells and extracellular vesicles, including short in vivo retention, low utilization, and transient therapeutic effects, various biomaterials have been extensively explored as delivery vehicles. These carriers can prolong the residence time of active components at target sites, maintain their biological activity, and enable controlled release. Consequently, they have emerged as a pivotal focus in tissue engineering and regenerative medicine.

### Hydrogels

#### Decellularized Extracellular Matrix (dECM) Hydrogels

Decellularized extracellular matrix (dECM) is derived from biological tissues and can recapitulate the composition and architecture of native ECM, providing a suitable microenvironment for cellular activity. Tissue-specific dECM hydrogels can support follicular development and promote the proliferation and differentiation of endometrial organoids. In addition, they serve as local delivery platforms for growth factors or PRP, enabling controlled release at target sites [82].

A study evaluated the effects of human umbilical cord blood-derived PRP alone versus its combination with porcine dECM hydrogel. PRP monotherapy increased endometrial area and glandular density, reduced collagen deposition, enhanced the cellular proliferation index, and upregulated regenerative factors such as Akt1 and VEGF. Although the composite hydrogel demonstrated favorable outcomes in tissue regeneration parameters, it did not produce a significant improvement in pregnancy rates, potentially due to suboptimal PRP concentration within the hydrogel. These findings suggest that while dECM hydrogels can enable sustained release of growth factors, their therapeutic efficacy requires further optimization [83].

A preclinical study employed decellularized endometrial (dECM) scaffolds derived from rat endometrium to repair surgically created circular defects in the rat uterus. The scaffolds, either loaded with silicone tubes or used alone, were implanted into the recipient uterine tissue. The regenerated regions within the scaffolds exhibited a richer endometrial stroma; however, complete reconstruction of the luminal and glandular epithelia was not achieved, indicating that additional interventions are required to promote full epithelial restoration [84].

The clinical translation of dECM hydrogels must consider two potential risks. First, immunogenicity: xenogeneic (e.g., porcine) or allogeneic dECM may retain residual antigens (e.g., α-Gal), DNA fragments, or intracellular proteins. These remnants could trigger host macrophage-mediated chronic inflammation or fibrotic encapsulation, potentially exacerbating adhesion formation [85]. Currently, systematic studies are lacking that compare the effects of different decellularization protocols—such as SDS, Triton X-100, or enzymatic digestion—on the immunogenicity of endometrium-specific dECM [86].

A second concern involves the mismatch between dECM scaffold degradation and tissue regeneration rates. If the scaffold degrades too slowly in vivo, residual material may physically obstruct glandular openings, hinder menstrual outflow, or serve as a nidus for fibrin deposition, potentially inducing re-adhesion. Ideally, dECM materials should exhibit “on-demand” degradability, meaning they are fully absorbed once the newly regenerated endometrium has substantially remodeled, typically within 2–4 weeks. Existing studies have demonstrated that the crosslinking density of dECM directly influences its in vivo degradation rate and regenerative performance [87]. Nevertheless, precisely regulating the crosslinking density of dECM to align with the cyclical regenerative rhythm of the endometrium—and even with individual patients’ hormonal cycles—remains a critical technical challenge.

#### Other Hydrogel Systems

Beyond dECM, various natural and synthetic polymers have been widely explored as delivery platforms for supporting endometrial repair.

Peptide-based hydrogels can encapsulate MSC-derived exosomes preconditioned in an inflammatory environment (IL-1β), enabling localized, gradual, and sustained release. These hydrogels exert anti-inflammatory effects by downregulating HMGB1 protein and the phosphorylated forms of IκB-α and p65, thereby promoting endometrial regeneration [88].

Alginate-based hydrogels can be crosslinked via calcium ions. One study developed a dual-network hydrogel composed of phenylboronic acid-functionalized polyethyleneimine and oxidized alginate, offering a novel approach for smart biomaterial applications [89]. Fang et al. engineered a dual-crosslinked alginate/recombinant collagen hydrogel, which exhibited favorable biocompatibility, injectability, and an appropriate degradation rate. This hydrogel enhanced endometrial stromal cell viability and maintained hormonal homeostasis, thereby accelerating stromal regeneration and structural reconstruction following uterine injury [90]. Encapsulation of decidual stromal cell-derived exosomes within alginate hydrogels enabled both sustained release over approximately five days and retention in the murine uterus for at least one week. This approach outperformed single-modality treatments in inhibiting fibrosis, promoting endometrial regeneration, improving vascularization, and enhancing endometrial receptivity and fertility restoration [91]. Xie et al. incorporated platelet-rich plasma into a composite hydrogel composed of polyethylene glycol diacrylate, alginate, and L-serine. L-serine facilitated rapid gelation and enhanced mechanical properties, while alginate contributed to the strength, toughness, and controlled degradability of the hydrogel, thereby achieving dual functions of growth factor delivery and physical barrier support [92].

Hyaluronic acid (HA) hydrogels have gained clinical utility as physical barriers due to their anti-inflammatory and pro-angiogenic properties, reducing the incidence of intrauterine adhesions and enhancing postoperative pregnancy outcomes [93]. Zhang et al. developed an injectable hydrogel composed of oxidized hyaluronic acid and gelatin grafted with hydrazide, which exhibited favorable cytocompatibility with human UCMSCs and effectively promoted endometrial regeneration in a rat model [94]. The tHA-tChi hydrogel, owing to its injectable, self-healing, and antioxidant properties, effectively scavenges reactive oxygen species (ROS) in the injured endometrium and ameliorates the local oxidative stress microenvironment. Acting as a dual sustained-release platform for platelet-rich plasma (PRP) and adipose-derived stem cells (ADSCs), it continuously delivers PRP-derived growth factors such as VEGF, PDGF, and EGF while providing a supportive niche for ADSCs. This system activates the VEGF/VEGFR2/AKT/BAD signaling pathway, thereby promoting endothelial cell proliferation, tube formation, and anti-apoptotic effects. Consequently, it enhances endometrial angiogenesis, reduces fibrosis, and increases gland number and endometrial thickness. In a mouse model of thin endometrium, this approach effectively restores embryo implantation capacity and the number of gestational sacs, offering a promising regenerative strategy for endometrial injury-related infertility [95].

Poloxamer hydrogels have been utilized to enhance the retention of therapeutic agents within the uterine cavity. Lin et al. encapsulated UCMSC-derived exosomes within a poloxamer hydrogel, which prolonged their intrauterine residence and mitigated fibrosis. This effect was achieved by downregulating fibrotic markers such as vimentin and COL5A2. This approach effectively restored both the structure and function of the damaged endometrium [96]. Zhang and colleagues developed a thermosensitive injectable Poloxamer-407 hydrogel co-loaded with KGF-2 and NO-MBs. In vitro studies and a rat model of ethanol-induced endometrial injury showed that the hydrogel enabled sustained release of both factors. KGF-2 and NO acted synergistically to upregulate VEGF and CD31, thereby promoting angiogenesis; enhance CK-18 and Ki67 expression to drive epithelialization and cell proliferation; reduce Caspase-3 levels to inhibit apoptosis; lower IL-6, TNF-α, and CD68-positive cells while increasing IL-10 to exert anti-inflammatory effects; and raise the Collagen-III/I ratio to maintain extracellular matrix homeostasis and reduce fibrosis. Collectively, the hydrogel significantly restored endometrial thickness, gland number, and structural integrity, increasing the pregnancy rate to 50% and the average embryo count to 5.3, demonstrating a clear ability to repair fertility [97].

Chen et al. [98] developed a hemispherical hydrogel composed of porcine-derived decellularized extracellular matrix (dECM) and methacrylated gelatin to deliver MenSCs. This composite hydrogel prolonged the intrauterine retention of the stem cells, resulting in a 2.3-fold increase in endometrial thickness and a 3.7-fold increase in glandular density. Fibrosis was attenuated through downregulation of α-SMA. Meanwhile, angiogenesis and endometrial receptivity were enhanced, as evidenced by the upregulation of CD31, VEGF, estrogen and progesterone receptors, vimentin, integrin β3, and leukemia inhibitory factor. Inflammation was concurrently suppressed, evidenced by decreased TNF-α levels. Structural and functional restoration of the endometrium was achieved. Specifically, the treatment group exhibited an average of 11.0 ± 2.8 gestational sacs, markedly exceeding that of the stem cell-only group (5.3 ± 1.2) and approaching normal values (12.3 ± 1.7). These findings demonstrate both robust reparative efficacy and clear translational potential. However, it is important to note that this study was conducted in a rat model, and the translational relevance to human endometrium—which has a different thickness, hormonal cycling, and regenerative capacity—requires cautious interpretation. Furthermore, the long-term safety of this composite hydrogel in terms of immunogenicity and degradation product clearance has not been evaluated.

Chitosan hydrogels, derived from natural polysaccharides, exhibit favorable biocompatibility, biodegradability, antimicrobial activity, and can be readily chemically modified. In one study, L-serine and allyl-functionalized chitosan were formulated into an injectable hydrogel loaded with PRP. This system allowed rapid gelation, with chitosan conferring injectability, flexibility, and mechanical robustness. The hydrogel reduced collagen deposition and promoted endometrial cell proliferation, ultimately restoring fertility in rats [99]. Another investigation incorporated extracellular vesicles into a thermosensitive chitosan/glycerophosphate hydrogel. A 2.0% chitosan concentration provided optimal porosity, rheological behavior, viscoelasticity, and self-healing capacity. This enabled sustained vesicle release for over seven days and enhanced their accumulation at the injury site, which substantially improved regenerative outcomes [100].

### Other Biomaterial Scaffold Systems

Collagen, a principal structural component of the extracellular matrix, exhibits high biocompatibility, biodegradability, and widespread availability, making it a widely used scaffold material in tissue engineering. It provides mechanical support while modulating cellular behavior. In one study, recombinant humanized type III collagen with enhanced cell-adhesive properties was applied in an in vitro model, successfully inducing the polarization of pro-inflammatory M1 macrophages toward a reparative M2 phenotype. This intervention improved the migration, viability, and collagen production of endometrial stromal cells under inflammatory conditions, while attenuating inflammatory responses and promoting extracellular matrix remodeling. These findings collectively suggest a potential strategy for restoring fertility [101].

Microneedle systems represent an emerging strategy for precise delivery. Li et al. [102] developed an antimicrobial microneedle platform loaded with endometrial cell spheroids, enabling minimally invasive, site-specific, and controlled release. This system stimulated angiogenesis through activation of the VEGF/VEGFR2 pathway, promoted epithelial and glandular regeneration via the Wnt/β-catenin pathway, and suppressed fibrosis through the TGF-β/Smad and Hippo/YAP pathways, thereby restoring tissue tension. Additionally, it modulated the immune microenvironment by influencing macrophage polarization and recruiting regulatory T cells. In a rat model, this approach achieved robust endometrial regeneration and functional fertility recovery. While conceptually innovative, the microneedle system’s actual delivery efficiency within the enclosed, humid, and dynamic uterine environment of humans remains unconfirmed. Issues such as needle dissolution kinetics, payload release profiles in the presence of uterine fluid, and patient acceptability (potential discomfort during application) have not been adequately addressed. Furthermore, clinical translation would require demonstration of safety in terms of endometrial perforation, infection risk, and long-term effects on endometrial architecture.

### Challenges and Perspectives

Despite ongoing advancements in biomaterial technologies, several challenges persist. Natural materials may exhibit batch-to-batch variability and potential immunogenicity, whereas degradation products of synthetic materials can elicit local inflammatory responses. Mismatches between scaffold degradation rates and tissue regeneration dynamics can compromise repair outcomes, as overly rapid or slow degradation may be detrimental. Scaffold fabrication remains complex, and issues such as batch consistency, long-term stability, and quality control continue to impede clinical translation. Conventional scaffolds primarily provide passive mechanical support and often lack the capacity to actively respond to abnormal microenvironmental cues within the injured tissue, such as inflammation or oxidative stress. Additionally, achieving truly personalized scaffold designs tailored to patient-specific injury profiles, hormonal status, or other physiological factors remains challenging.

A particularly underappreciated challenge is the mismatch between the degradation kinetics of biomaterials and the cyclical regenerative dynamics of the human endometrium. During reproductive years, the human endometrium undergoes complete shedding and regeneration approximately every 28 days. Most currently designed hydrogels and scaffolds have degradation profiles ranging from days to weeks, but they are typically evaluated in animal models that do not recapitulate human menstrual cycling. Whether a scaffold that works in a rat, an animal that does not menstruate, will perform similarly in humans remains unknown. Furthermore, if a scaffold degrades too slowly, it may persist through multiple cycles and interfere with normal endometrial shedding and regeneration. If it degrades too rapidly, its therapeutic benefit may be lost before adequate repair occurs. Achieving timed, synchronized degradation that respects the menstrual cycle remains an unresolved engineering challenge.

Future research should focus on the development of bioresponsive intelligent scaffolds capable of on-demand therapeutic release and dynamic modulation. Material degradation kinetics should be optimized to achieve synchronization with tissue regeneration processes. Standardized fabrication protocols and rigorous quality control systems are necessary to ensure product consistency. Bridging fundamental research with clinical studies, combined with long-term follow-up, is essential to validate safety and efficacy. These efforts will ultimately facilitate the implementation of individualized, precision-guided regenerative therapies.

We summarized 15 clinical trials comparing the efficacy of stem cells (BMMSCs, UCMSCs, EnSCs, ADSCs) and PRP in endometrial repair (Table 3).

**Table 3:** Clinical trial summary

Treatment method	Sample size	Endometrial thickness improvement (mm)	Pregnancy / Live birth	Study type	Limitations
Autologous BMMSC transplantation [23]	1	3.6 to 8.0	Successful pregnancy	Case report	First reported case, patient with severe IUA
CD133⁺ BMMSC transplantation [24]	11	IUA group: 4.3 to 6.7; Atrophic endometrium group: 4.1 to 5.5	5 pregnancies	Prospective study	Significant short-term effect, returned to baseline after 6 months
BMMSC infusion [25]	25	3.3±1.0 to 5.1±1.9	3 pregnancies, 2 live births	Prospective study	Small sample size, lost to follow-up
Autologous bone marrow nucleated cell transplantation [26]	20	4.3±1.1 to 6.2±1.4	5 pregnancies, 1 live birth	Prospective study	Small sample size, no control group, 1-year follow-up
Autologous BMMSC intrauterine infusion [27]	78	Single infusion: 4.3±1.2 to 6.4±1.5; Triple infusion: 4.1±1.1 to 7.2±1.6	Single infusion: 10 pregnancies, 6 live births; Triple infusion: 17 pregnancies, 12 live births	Retrospective design	2-year follow-up, diminished effect after single infusion
UCMSCs + collagen scaffold [38]	18	4.08 to 5.87	4 pregnancies, 2 live births	Self-controlled study	Small sample size, single-center study, 1-year follow-up
UCMSCs + collagen scaffold [39]	24	Treatment group: 5.3±0.7 to 6.5±1.3	Treatment group: 5 pregnancies, 3 live births	Double-blind RCT	Small sample size, short follow-up, higher rate of placenta accreta
Autologous EnSC intrauterine infusion [46]	15	4.2±1.1 to 6.8±1.3	9 pregnancies, 8 live births	Self-controlled study	Small sample size, no control group, 1-year follow-up
Autologous ADSC transplantation [47]	5	3.0±1.0 to 6.9±2.9	1 pregnancy (miscarriage at 9 weeks)	Small sample study	Requires validation with larger sample size
PRP intrauterine infusion [54]	40	5.2 to 7.8	19 pregnancies	Small sample study	Short follow-up, live birth rate not assessed
PRP subendometrial injection [55]	220	Study group: 5.8±0.9 to 7.7±1.2	Study group: 64 pregnancies, 48 live births; Control group: 36 pregnancies, 24 live births	Controlled study	Non-randomized control, single-center study
PRP intrauterine infusion [56]	220	Significant increase (P=0.037)	Treatment group: 42 live births	Retrospective study	Followed until delivery, non-randomized grouping, single-center study
PRP intrauterine infusion [61]	100	Single infusion: 7.96±0.45; Double infusion: 8.42±0.53	Single group: 10 pregnancies; Double group: 22 pregnancies	Controlled study	Single-blind design, single-center study, limited inclusion criteria

## Conclusions

Endometrial repair has evolved from conventional hormonal and surgical interventions to a regenerative medicine paradigm that integrates cell-based therapies, acellular approaches, and bioengineering strategies. Therapies such as mesenchymal stem cells, their derived extracellular vesicles, and platelet-rich plasma modulate key signaling pathways involved in fibrosis, inflammatory responses, and angiogenesis. In preclinical models and early-phase clinical studies, these interventions have demonstrated potential to enhance endometrial regeneration, improve receptivity, and support favorable pregnancy outcomes.

Despite the promising prospects of regenerative approaches for endometrial repair, several significant challenges hinder their clinical translation:

### Transition from Broad-Spectrum Repair to Precision Intervention

While traditional therapies focus on generalized anti-fibrotic or anti-inflammatory strategies, single-cell transcriptomic insights have identified more specific therapeutic targets. A pivotal example is the recent identification of the GZMA-PARD3 signaling axis in thin endometrium [10]. Unlike generic inflammation markers (e.g., IL-6 or TNF-α) that merely reflect tissue damage, the suppression of the GZMA-PARD3 axis directly drives the depletion of IGFBP3 + Stromal-1 cells—the key progenitor population responsible for functional endometrial regeneration. Mechanistically, impaired GZMA-PARD3 signaling disrupts cell polarity and tight junction assembly, shifting the stromal niche from a regenerative to a profibrotic phenotype. This explains why broad-spectrum therapies (such as unmodified MSCs or PRP) often fail: they may reduce inflammation but do not reverse the specific molecular blockade preventing stromal progenitor renewal. Consequently, future precision interventions should aim to restore this specific axis, potentially through engineered extracellular vesicles delivering recombinant GZMA or targeted gene editing, rather than relying on nonspecific cellular proliferation signals.

### The Mechanistic Understanding of Combination Therapies Lags Behind their Observed Efficacy

Existing studies have explored various combination strategies, such as stem cells with hydrogels or platelet-rich plasma with scaffolds. Most investigations, however, remain confined to phenotypic observations and do not clarify the temporal and spatial contributions of individual components. For instance, it remains uncertain whether the biomaterial first modulates the immune microenvironment and then the stem cell-derived factors repair parenchymal cells, or if these processes occur concurrently. This “black box” characteristic of combination therapies constrains rational design. Future research should integrate in vivo imaging, spatial omics, and systems biology models to dissect rate-limiting steps and identify molecular switches that govern the synergistic effects.

### Limitations of Current Clinical Evidence

A critical limitation of existing literature is the disconnect between surrogate endpoints and patient-centered outcomes. Among the 18 clinical studies included in this review, 14 (77.8%) employed endometrial thickness as the primary endpoint, and 11 (61.1%) reported statistically significant increases in thickness (mean Δ = 1.8 ± 1.1 mm). However, only 6 studies (33.3%) observed a significant improvement in live birth rate, and no study has yet established a dose-response relationship between the magnitude of thickness gain and live birth probability. Notably, in two bone marrow-derived mesenchymal stem cell (BMMSC) trials, endometrial thickness—despite showing significant initial increases—returned to baseline levels by 12 months post-intervention, without corresponding improvements in live birth rates [26, 29].

Furthermore, the majority of current clinical evidence carries a high risk of bias. Of the eight randomized controlled trials (RCTs) assessed, seven (87.5%) were rated as “high risk” or “some concerns” according to the Cochrane RoB 2 tool. Major methodological flaws centered on selection bias (inadequate reporting of randomization and allocation concealment) and performance bias (lack of blinding in interventions such as PRP administration). Crucially, there was a prevalent lack of complete outcome data for the primary endpoint of live birth, and several trials were suspected of selective reporting.

Moreover, the current evidence base is heavily skewed toward low-quality observational designs (Supplementary Table 1). All non-randomized studies were deemed to have a “serious risk of bias,” primarily attributable to confounding and selection effects, as these studies generally lacked control groups or failed to adjust for baseline endometrial pathology. This hierarchy-of-evidence gap—where claims of efficacy are underpinned by short-term, high-bias data—necessitates extreme caution when interpreting current clinical success rates.

### A Further Challenge Lies in the Insufficient Dynamic Adaptability of Current Biomaterial Designs

Issues related to immunogenicity, degradation kinetics, and mismatch with the endometrial regenerative cycle remain unresolved. Ideal carriers should exhibit “on-demand” degradability while actively modulating the pathological microenvironment to promote tissue repair. Achieving degradation profiles that synchronize with the menstrual cycle (approximately 28 days) rather than with arbitrary time points from animal studies is a critical unmet need.

### A Notable Bias Exists in the Anchoring of Clinical Endpoints

Numerous studies continue to use endometrial thickness as a surrogate for treatment success, whereas patient-centered outcomes such as live birth rates and long-term offspring health remain largely unreported. This is problematic because improvements in endometrial thickness do not consistently translate into improved live birth rates, as evidenced by several studies that reported increased thickness without corresponding pregnancy success. Regulatory authorities and professional societies should promote the adoption of patient-focused endpoints—including cumulative live birth rate, miscarriage rate, and pregnancy complications—as primary benchmarks for efficacy assessment.

In summary, endometrial regenerative medicine can transition from an “experimental breakthrough” to a “clinical standard” only by directly addressing these deep-seated challenges: rigorous clinical trials, standardized manufacturing and quality control systems, and interdisciplinary collaborative innovation. Such advancement will ultimately provide higher-level evidence to guide clinical decision-making and yield tangible benefits for patients.

## Supplementary Information

Below is the link to the electronic supplementary material.

Supplementary Table 1 (DOCX 31.0 KB)

## Data Availability

No new data were generated or analyzed in this review. Data sharing is not applicable to this article.
