# Supplementary material for: Endometrial Repair and Regenerative Medicine: From Broad-Spectrum Regeneration to Precision Intervention
Source: Reprod Sci. 2026 Jul 27;33(9):1867–89. doi: 10.1007/s43032-026-02150-3 (PMC13562326; doi:10.1007/s43032-026-02150-3)
Supplement: Supplementary file 1 [file 43032_2026_2150_MOESM1_ESM.docx]

**Supplementary Table1. Quality Assessment Table of Key Clinical Trials**

|  | **Therapy** | **Study design** | **Primary endpoint** | **Follow-up duration** | **Level of evidence** | **Main limitations** |
| --- | --- | --- | --- | --- | --- | --- |
|  | Autologous BMMSC | Case report | Endometrial thickness, pregnancy | Not specified | IV | Single case, not generalizable |
|  | CD133⁺ BMMSC | Prospective, non-controlled | Endometrial thickness, vascular density | 6 months | III | Effect disappeared after 6 months, small sample size, no control |
|  | BMMSC infusion | Prospective | Endometrial thickness | 5 years | III | Possible loss to follow-up, single-arm longitudinal study |
|  | Autologous ADSC | Prospective | Endometrial thickness | Not specified | IV | Small sample size, no live birth |
|  | UCMSC + collagen scaffold | Self-controlled, prospective | Endometrial thickness, uterine receptivity | Until delivery | III | No concurrent control |
|  | Autologous bone marrow mononuclear cells | Prospective, experimental | Endometrial thickness, pregnancy | 1 year | III | Non-controlled study, live birth rate 5% |
|  | Lyophilized PRP | Small-sample study | Endometrial thickness, pregnancy | Not specified | III | Small sample, live birth not reported |
|  | Autologous BMMSC + collagen scaffold | RCT | Ongoing pregnancy rate, live birth rate | Partially until delivery | II | Single center; increased incidence of mild placenta accreta |
|  | UCMSC + collagen scaffold | Double-blind RCT | Pregnancy rate, live birth rate | Partially until delivery | II | Small sample size |
|  | Autologous EnSC | Self-controlled | Pregnancy rate, live birth rate | 2 years | III | No control group, combined intervention |
|  | PRP (subendometrial injection) | Prospective cohort | Endometrial thickness, pregnancy | Not specified | III | Non-randomized |
|  | PRP (intrauterine infusion) | Retrospective cohort | Endometrial thickness, pregnancy, live birth rate | Partially until delivery | II-III | Non-randomized grouping, single center, retrospective design |
|  | PRP (single vs. double) | RCT | Endometrial thickness, pregnancy rate | Short | II | Live birth not reported, exclusion of RIF/RPL patients |

Note: RCT, randomized controlled trial; RIF, recurrent implantation failure; RPL, recurrent pregnancy loss.
